# Effect of Silicate Slag Application on Wheat Grown Under Two Nitrogen Rates

**DOI:** 10.3390/plants6040047

**Published:** 2017-10-11

**Authors:** Brandon White, Brenda S. Tubana, Tapasya Babu, Henry Mascagni, Flavia Agostinho, Lawrence E. Datnoff, Steve Harrison

**Affiliations:** 1 School of Plant, Environmental, and Soil Sciences, Louisiana State University AgCenter, Baton Rouge, LA 70803, USA; B17white@hotmail.com (B.W.); Fagos2@lsu.edu (F.A.); SHarrison@agcenter.lsu.edu (S.H.); 2 Compass Minerals Innovation Center, Stilwell, KS 66085, USA; BabuT@compassminerals.com; 3 Northeast Research Station, Louisiana State University AgCenter, St. Joseph, LA 71366, USA; HMascagni@agcenter.lsu.edu; 4 Department of Plant Pathology & Crop Physiology, Louisiana State University AgCenter, Baton Rouge, LA 70803, USA; LDatnoff@agcenter.lsu.edu

**Keywords:** silicate slag, silicon, nitrogen, wheat, grain yield, soil pH, plant-essential nutrients

## Abstract

Field studies were established on the alluvial floodplain soils in Louisiana, from 2013 to 2015, to evaluate the effect of silicate slag applications on productivity of wheat (*Triticum aestivum*), under sufficient and high nitrogen (N) application rates. Treatments were arranged in a randomized complete block design, with four replications consisting of twelve treatments: a factorial combination of two N (101 and 145 kg N ha^−1^) and five silicate slag rates (0, 1, 2, 4.5, and 9 Mg ha^−1^), and two control plots (with and without lime). Nitrogen had a greater impact on wheat productivity than silicate slag application. Wheat grain yield reached over 7000 kg ha^−1^ with applications of 145 kg N, and 9 Mg silicate slag per ha for soil having Si level <20 mg kg^−1^. Yield increases due to N or Si were attributed to the increase in number of spike m^−2^ and grain number spike^−1^. Silicate slag application effectively raised soil pH, and availability of several plant-essential nutrients, including plant-available N (nitrate, NO_3_^−^), demonstrating the benefits of slag application are beyond increasing plant-available Si. The benefits of silicate slag application were clearly observed in wheat supplied with high N, and on soil with low plant-available Si.

## 1. Introduction

Wheat is one of the most important crops worldwide, with global production exceeding that of all other crops [1]. More nourishment has been received from wheat for the world’s population than any other food grain [2]. The United States is one of the largest global producers of wheat, and ranked third in planted area during the first 10 years of the 2000s [3]. Wheat production area occupies about 60,000 ha in Louisiana with an estimated total value of $75 million in 2014 [4]. The high annual rainfall and high temperatures in Louisiana challenge wheat productivity through high pest and disease pressure, poor N utilization, and short grain fill periods [5]. Silicon (Si) is a beneficial plant nutrient that has been shown to improve yields in a variety of crops, especially members of Poaceae family [6]. Although wheat may accumulate close to 4% Si in plant tissue, research on this crop using Si lags far behind other crops, such as rice (*Oryza sativa*) and sugarcane (*Saccharum officinarum*) [7,8].

Silicon is prevalent in the soil, but primarily exists as silica (SiO_2_), which is not available for plant uptake. Silicon must be taken up by plant roots in the form of mono-silicic acid (H_4_SiO_4_), and the natural dissolution of SiO_2_ to H_4_SiO_4_ in the soil is slow [9]. Upon uptake, Si is deposited as amorphous silica (SiO_2_·nH_2_O) or opal phytoliths in cell lumens, cell walls, and intercellular spaces [9,10]. Once it is deposited, SiO_2_ is not redistributed within the plant [6]. The strengthening of these protective layers and the increase in overall structural integrity is one hypothesis for a number of benefits associated with Si uptake in plants. Silicon has been shown to increase resistance to multiple biotic and abiotic stresses, such as lodging, disease, and pest damage [7,11,12]. Positive responses of plant growth parameters to Si fertilization have been observed. Ma et al. (1989) reported increases in the number of panicles, spikelets per panicle, and decreases in the number of blank spikelets when Si was applied [13]. Increases in grain weight were also observed, as well as plant height and longer spikes in wheat [14,15]. These and other benefits of Si fertilization may contribute to yield increases.

Highly weathered, leached or organic soils, such as Histosols, are commonly deficient of available Si [16]. Soils planted to Si-accumulating crops can also diminish Si levels, furthering the potential responses to Si fertilization [17,18]. Silicate slags are common sources of Si, and are by-products from the steel manufacturing industry as well as from elemental phosphorus (P) production [19,20]. Silicon fertilization has become a common practice contributing to higher yields in crops such as rice and sugarcane [6]. The use of slags is widespread in Japan for degraded paddy soils in rice production [20]. Yoshida (1981) reported that yield increases of 10% are common in these and similar areas, and when leaf blast is severe, yield increases up to 30% were observed [21]. Using silicate slags, Korndorfer et al. (2001) reported yield increases in 19 out of 28 field experiments in rice production in the Everglades Agricultural Area in south Florida [22]. In a study conducted by Raid et al. (1992), sugar yield of cane applied with 6.7 Mg ha^−1^ were between 17.2 and 21.8% higher than the untreated cane for two successive cropping years [23]. Korndorfer et al. (2001) established 4.5 Mg ha^−1^ as an optimum rate for rice production, but responses in yield have been observed at up to 15 Mg ha^−1^ [22]. While higher rates are sometimes needed to reach greater yield increases, this is not always the case. Ma and Takahashi (2002) reported somewhat lower rates of 1.5 to 2 Mg ha^−1^ as common for many areas in Japan [19]. Like with other plant nutrients, fertilizer application rates can vary depending on several factors, including soil and crop type, as well as existing soil nutrient status.

Since N is the most limiting plant nutrient in non-leguminous crop production systems, and therefore, the most often applied among other nutrients, it is important to understand the relationship between Si and N. To prevent deficiencies, producers typically apply N in excess of the crop requirement. Excessive applications of N fertilizer can also result from not using effective N decision tools. Overuse of N fertilizers in agriculture is a non-point source of pollution to surface and groundwater systems [24]. However, excessive N application can also result in lodging, and increases in pest and disease damage [25,26,27], eventually reducing yield and income. These effects could be potentially minimized by the use of Si. Silicon has been reported to raise the optimum level of N in rice. Ho et al. (1980) reported that due to a synergistic effect, the application of Si has the potential to raise the optimum N rate use efficiency, thus enhancing productivity of existing lowland rice fields. Silicon has been reported to raise the optimum level of N in rice [28]. The interaction between Si and N fertilizer has been evaluated in crops like rice and sugarcane [12,29]. A study of the effects of N and Si nutrition on the susceptibility of sugarcane to the African sugarcane borer (*Eldana saccharina*) by Meyer and Keeping (2005) not only showed that Si reduced the susceptibility to the pest across multiple N rates, but that tissue N/Si ratios were more correlated with resistance to *E. saccharina* than N or Si alone [12]. A study by Wallace in 1989 found that as the N supply to a monocot species increased, the uptake of Si decreased [30].

While the benefits of Si in wheat have been documented, the combined effects of N and Si fertilization have not been evaluated, particularly in Louisiana. Therefore, this study was conducted to determine the impact of silicate slag application on grain yield of wheat under sufficient and high N application rates. The changes on N and Si uptake, biomass yield, soil pH, and plant-essential nutrient content of the soil, were also documented.

## 2. Results

The N, Si, and N × Si interaction effects on grain yield, N uptake, and Si uptake for all three site-years are reported in Table 1. Straw yield was only impacted by N, and only at Central Research Station in Ben Hur (BH) in 2014. A significant N × Si interaction effect on straw yield was observed at Northeast Research Station (NERS) in 2014. Nitrogen had a significant effect on grain yield at all three site-years, while a Si effect was only observed at BH in 2014 (*p* < 0.05). There was no significant interaction effect detected between N and Si on grain yield. Nitrogen had no recorded effect on straw N uptake except at BH in 2014, whereas for grain N uptake, both N and Si effects were significant at two of the three site-years. There was no noticeable effect of N × Si on Si uptake (straw, grain and total), except at NERS in 2014.

**Figure 1:**
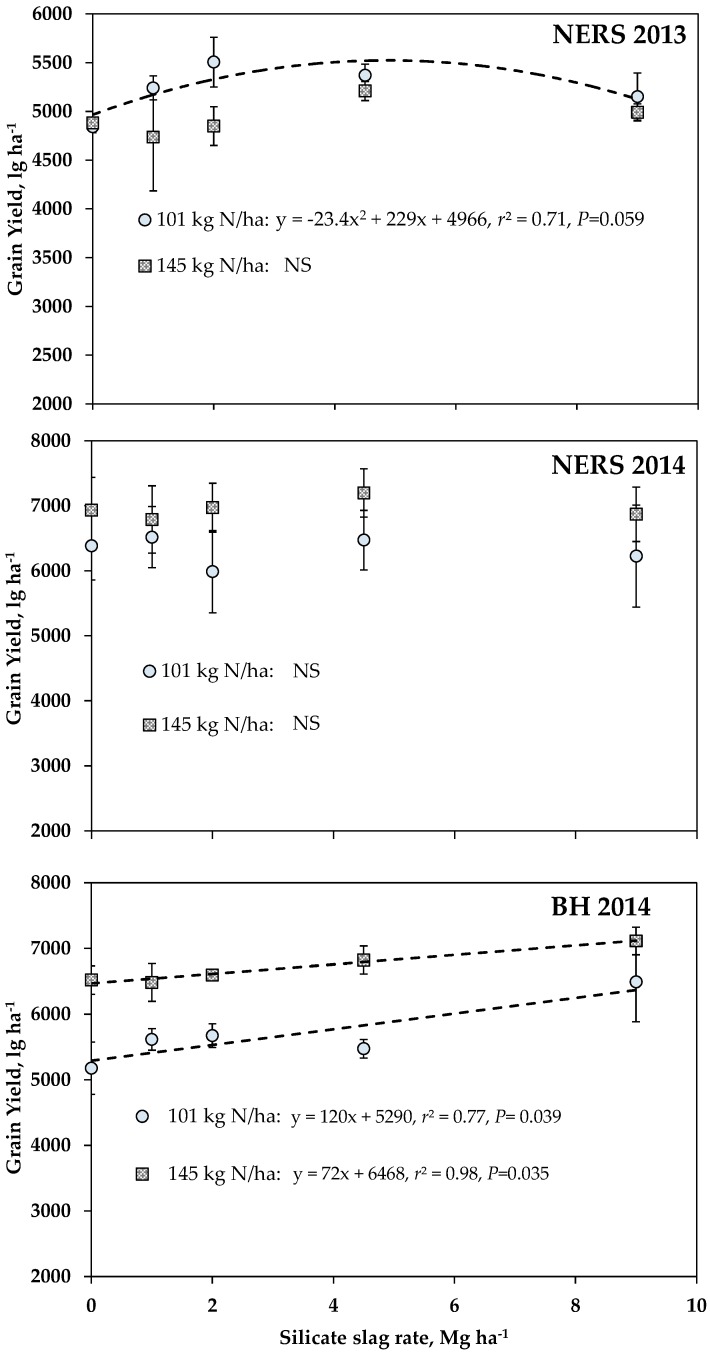
Grain yield of wheat applied with varying rates of silicate slag under sufficient and high N application rates NERS 2013, NERS 2014, and BH 2014. NS denotes no significant effect on yield; P values reported are for the regression model between yield and silicate slag for each N rate.

Trend analysis between grain yield and silicate slag rates for each level of N for each site-year is shown in Figure 1. This was also done to grain yield response of wheat with increasing silicate slag rate for each level of N. Higher grain yields were recorded for plots which received 145 kg N ha^−1^ at NERS in 2014 and BH in 2014. At BH in 2014, grain yield linearly increased with silicate slag rate, reaching >7000 kg ha^−1^, level with the application of 9 Mg ha^−1^ silicate slag, whereas at NERS in 2013, the application of silicate slag at 2 Mg ha^−1^ improved yield, but for plots with 101 kg N ha^−1^. At NERS in 2014, grain yield between 101 and 145 kg N ha^−1^ treatments were similar. The application of 2 Mg ha^−1^ silicate slag to plots which received 101 kg N ha^−1^ resulted in a significant increase in grain yield by >500 kg ha^−1^ (Figure 1). The results from the analysis of variance (ANOVA) on yield components showed that the impact of N, Si, and N × Si interaction was not consistently observed for all the three site-years (Table 2, Table 3 and Table 4). A general trend was that wheat applied with higher N rate tended to have higher number of spikes m^−2^. At NERS in 2013 and in 2014, the interaction effect of N and Si was significant (*p* = 0.08) if the level of confidence was set at 0.1. Here, the optimal silicate slag rate to attain the highest spike count was different for each N rate, i.e., 9 Mg ha^−1^ for 145 kg N ha^−1^, and between 2 to 4.5 Mg ha^−1^ for 101 kg N ha^−1^ (Table 2 and Table 3). Similarly, the effect of Si on grain count per spike was only observed in plots applied with 145 kg N ha^−1^. Overall, the higher number of spike m^−2^ and grain count per spike contributed to differences in grain yield observed in this trial. The average spike length and width did not respond to N and Si treatments. The number of grain per spike tended to be higher in wheat applied with 145 kg ha^−1^, than those with 101 kg N ha^−1^. The grain analysis showed that N consistently affected %N and %protein in grain with 145 kg N ha^−1^ having higher values than those applied with only 101 kg N ha^−1^. In addition, Si treatment impacted %N and %protein at NERS in 2014 and BH in 2014. Plots treated with silicate slag generally had higher N and protein content than plots without silicate slag. On the other hand, the lack of N and Si impact on Si content of grain was consistently observed across site-years.

Silicate slag application influenced soil pH and nutrient content more than the N application (Table 5, Table 6 and Table 7). Both soil pH and soil Si content based on 0.5 M acetic acid extraction procedure were increased with silicate slag application. Increasing silicate slag rate increased soil Si, with the highest increased by as much as 80 mg kg^−1^ (>150%) with the application rate of 9 Mg ha^−1^. Increases by at least 0.3 unit of pH were observed with a minimum of 2 Mg ha^−1^ application of silicate slag. With a 9 Mg ha^−1^ application rate of silicate slag, the highest increase in pH was 0.9 unit (Table 5 and Table 7). The application of silicate slag made a positive impact on the extracted amount of calcium (Ca), magnesium (Mg), S, manganese (Mn), Zn, and even copper (Cu) at NERS in 2014, based on Mehlich-3 extraction procedure. On the other hand, the amount of iron (Fe) extracted from the soil was reduced with an increasing rate of silicate slag (Table 7). Between ammonium (NH_4_^+^) and NO_3_^−^, silicate slag rate increased the amount of NO_3_^−^ extracted from the soil in all three site-years. Nitrogen treatment had an effect on the NH_4_^+^ and NO_3_^−^ content, being higher in plots which received 145 kg ha^−1^ than those with 101 kg N ha^−1^ application, but only at NERS in 2014 and BH in 2014.

## 3. Discussion

Results on grain yield from site-years at NERS in 2013 and BH in 2014 demonstrated that N had a higher impact on wheat grain yield than Si. This finding is not unexpected, since N is the most limiting nutrient in crop production, and it is common to obtain observable grain yield responses to N application rather than Si. Heavy rainfall was recorded around the time of N application, which could have resulted in loss of the N applied from the soil profile through leaching and a denitrification process. At NERS in 2014, the total amount of rain received was ~500 mm for the months of January and February. At NERS in 2013, an average of 180 mm rainfall was received in March and April following 100 mm rain received in February. This could partly explain the greater grain yield response to N observed at BH in 2014 (Figure 1). Cereal crops and sugarcane recovery of applied N fertilizers is low, ranging from only 20% to 40%, with ~65% presumably lost from the crop–soil systems [31,32,33,34]. In the present study, the NO_3_^−^ content of the soil was consistently lower than NH_4_^+^ across the site-years; this is associated with the mobility of NO_3_^−^ in the soil, being 5 to 10 times faster than NH_4_^+^ and other N forms, such as amino acids [35].

The high rainfall and subsequent leaching could have also led to the loss of applied Si from the plant root zones. This might also explain the lack of wheat grain yield response to Si application, even though the soil Si level was considered to be low (36 mg kg^−1^). The severe and frequent soil erosion and sediment transportation in areas owing to high rainfall and coarse texture of soil might also have led to desilication, and thus, a relatively low soil Si [36]. Among the site-years, BH in 2014 had the lowest initial soil Si level (17 mg kg^−1^); this soil Si level is below the initial critical soil Si level established for soils of Louisiana [37]. The effect of Si on yield was significant, with higher yields observed at the highest silicate slag rate at 9 Mg ha^−1^ for both N rates (Figure 1). It is notable that grain yield was the highest (~7000 kg ha^−1^) from plots which received 145 kg N ha^−1^ and 9 Mg ha^−1^ silicate slag, suggesting the important role of Si in minimizing the yield decline factors, like lodging and pest and disease damage that may have been caused by excessive N application [29]. Korndorfer et al. (2001) reported yield increases in rice with Si fertilization in several different field experiments [22]. Similar yield increases have also been reported for rice production in Japan [21] and in wheat [15,38]. While 9 Mg ha^−1^ was the highest yielding silicate slag rate for both N rates at BH in 2014, it is important to note that this high rate may limit the economic return and feasibility of application of silicate slag. Slag materials are by-products and relatively inexpensive compared to other common fertilizers, but transportation costs are expensive for fields that are quite far from slag manufacturers (or suppliers). Also, the amount of yield increase should be weighed out carefully to justify the cost of applying high rates of silicate slag. In addition, soils which have high pH may encounter nutrient-related problems as silicate slag is a potent liming agent [38], and at this rate, might cause precipitation of micronutrients, thus causing a deficiency in crops.

The pH, 0.5 M acetic acid extractable Si, soil NO_3_^−^ and NH_4_^+^, and Mehlich-3-extractable nutrients of soil sampled at harvest, are summarized in Table 5, Table 6 and Table 7 for each site-year. Silicate slag has a high liming potential [38], thus effective at increasing soil pH. As a by-product, silicate slag contains other elements, such as Ca, Mg, Mn, Fe, and S. The soil analysis showed that all of these nutrients, except for Fe, increased with the application of silicate slag. Silicon rates significantly increased the amount of Ca, Mg, Mn, and S in the soil, but this was expected because the silicate slag material used in these trials contains these elements. While the significant increases in S at NERS in 2013 and BH in 2014 can be attributed to the direct result of addition of silicate slag due to the presence of S in the material, the increase in pH may have also influenced the increase in soil S extracted. The soil pH at NERS in 2013 and BH in 2014 was increased by 0.9 units of pH with silicate slag application, whereas the soil pH of NERS in 2014 reached a maximum of only 5.5 (acidic). The increase in soil pH can considerably reduce the adsorption of sulfate ions (SO_4_^2−^) because adsorption of sulfate occurs on positively charged sites in a soil matrix that are pH dependent, and becomes negligible at pH values greater than 6 [39,40]. Similarly, the increase in addition of silicate slag could have resulted in an increase of the SiO_3_^2−^ ion competition for the anion sorption sites in soil. The reduction in pH dependent anion sorption sites in soils of NERS in 2013 and BH in 2014 due to the rise in pH up to ~7, combined with an increase in SiO_3_^2−^ ion competition for these sites in soil, could have drastically reduced the sorption of SO_4_^2−^ at these sites. Management practices, such as liming and phosphate additions, are known to release some of the adsorbed SO_4_^2−^ [41]. Therefore, increase in plant available S in soil could not only have been a direct but also an indirect result from the addition of silicate slag as well. On the other hand, the reduction in Mehlich-3 extractable Fe content of the soil at NERS in 2014 may likely be associated with Fe precipitation due to the increased soil pH. While Zn is not a component of the silicate slag material, its availability increased as Si rates increased for all three site-years (*p* < 0.05). These results contradict what was expected, as Zn availability decreases with increasing soil pH [42]. However, Zn availability still increased in these soils despite the rise in soil pH observed as silicate slag rates increased. These results disagree with those by Saleh et al. (2013) and Cunha et al. (2008), where Si applications lead to decreases in extractable Zn [43,44]. This finding could simply be due to pH, since in this study, it was never above 7. Although soil pH governs the speciation of Zn in solution, Zn^2+^ (plant available form) predominates at pH values below 7.7, and ZnOH^+^ is the main species at pH above 7.7, and the neutral species Zn(OH)_2_ is dominant only at pH above 9 [45]. The increased availability of Zn in soil could also be due to the increase in concentration of Ca and Mg in soil, consequent with the addition of slag. Zhu and Alva (1993) studied the relative effect of Ca, Mg, and K on the adsorption of Zn and Cu [46]. Adsorption of both Cu and Zn decreased with an increase in concentrations of either Ca or Mg. This reduction in adsorption was attributed to increased competition for exchange sites with increased ionic strength. They also reported that this inhibitory effect of added cations on metal adsorption was greater for Zn than Cu.

The percent organic matter of BH in 2014 was lower among the three site-years studied with NERS in 2013, NERS in 2014, and BH in 2014 having 2.3%, 2.1%, and 1.5% organic matter, respectively. It seems that in the soils of NERS 2013 and 2014, the solution concentration of Al was reduced by the sorptive properties of organic matter or the formation of Al ion complexes by organic matter (organic acids) [47,48,49,50,51,52,53,54]. Also, the initial concentration of Ca, Mg, and K, at NERS in 2013 and 2014, were greater than BH 2014, with only 1833, 481, and 146 mg kg^−1^ of Ca, Mg, and K, respectively. Zołotajkin et al. (2011) documented the presence of cations which compete with Al for exchange sites as an additional factor that might govern the concentration of exchangeable Al in the soil [55]. Similarly, it might be the presence of cations which compete with Al for exchange sites that resulted in lower concentration of exchangeable Al at NERS in 2013 and 2014. With comparatively lower concentration of these cations at BH in 2014 soil, it is likely that Al occupied the exchange sites over these basic cations. These basic cations, which are then present in soil solution, are easily lost from the surface soil due to various processes like leaching, runoff, and plant uptake over the period of crop growth. Whereas in the case of NERS in 2013 and 2014, the relatively higher concentration of these basic cations that compete for exchange sites with Al could have kept them in the surface soil for longer duration and at a higher concentration. However, this conclusion needs to be confirmed by further research.

With respect to agronomic responses, the application of N resulted in more consistent and significant changes in grain yield, yield components, and grain composition than silicate slag application. Increases in grain yield in plots which received higher N rate (145 kg N ha^−1^) were associated with higher number of spikes m^−2^ combined with higher numbers of grain per spike. The higher supply of N also improved %N and %protein content of grain. Previous studies showed similar effects on wheat yield components and quality [56,57]. However, there was no associated improvement on average spike width and length, or 1000 g weight with increased N rate. In fact, there was a tendency for these components to be lower in plots with 145 kg N ha^−1^ than plots with 101 kg N ha^−1^. This is consistent with the report of Barbottin et al. (2005) on the higher N remobilization observed in wheat under low N supply compared to high N supply [58]. On the impact of Si, it was only at BH in 2014 where the application of silicate slag application made a significant impact on grain yield (Figure 1), and this increase can be attributed to an increase in number of spikes m^−2^ (Table 4). The improvement in grain yield can also be tied up with the increase in soil NO_3_^−^ content, along with other essential nutrients, when silicate slag was applied. Between N and silicate slag, this study demonstrated the stronger influence of silicate slag application on maintaining an elevated level of NO_3_^−^ in the soil, as opposed to N application, and also, improvement in the levels of some plant-essential nutrients and soil pH.

## 4. Materials and Methods

**Figure 2:**
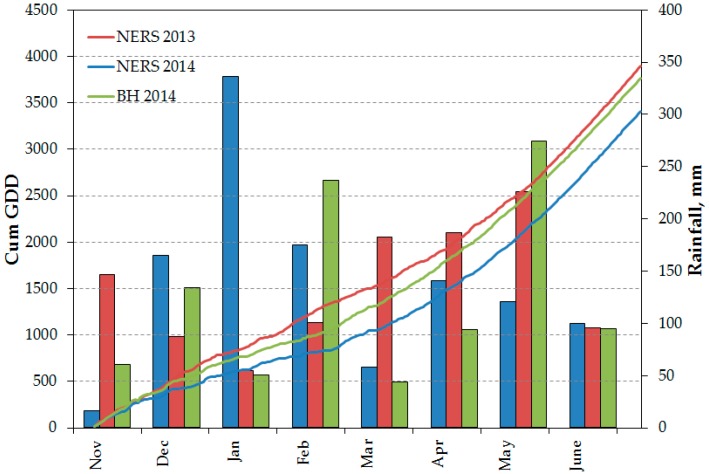
Cumulative growing degree days and monthly rainfall distribution from the beginning (November) and the end (June) of cropping season for each site-year.

This study was conducted from 2012 to 2014 with a total of three site-years. The first site-year was established in 2012 at St. Joseph (Latitude 31°56′42.6″ N; Longitude 91°13′34″ W), Louisiana on a Commerce silt loam soil (fine-silty, mixed, superactive, nonacid, thermic Fluvaquentic Endoaquepts). In 2013, this study was established at two locations: (1) NERS, St. Joseph, Louisiana (Latitude 31°56′41.0″ N; Longitude 91°13′25.8″ W) on a field with Sharkey–Tunica–Newellton complex (very fine, smectitic, thermic Chromic Epiaquerts) and Commerce silt loam soil types and (2) BH (Latitude 30°21′40.4″ N; Longitude 91°10′01.9″ W) near Louisiana State University campus in Baton Rouge on a Cancienne silt loam soil (fine-silty, mixed, superactive, nonacid, hyperthermic Fluvaquentic Epiaquepts). All of these soils are found on the alluvial floodplain along the Mississippi River. Figure 2 summarizes the cumulative growing degree days and monthly rainfall distribution during the cropping season for each site-year. Nitrogen fertilizer was applied in February, and among the site-years, Ben Hur in 2014 received the highest amount of rain. In addition, the rate of accumulation of positive growing degree days was slower in this site-year compared to NERS 2013 and 2014.

All three site-years were established using a dryland, conventional tillage system. Table 8 provides information on the locations, site-year ID and major field activities for the three site-years. Initial soil data for all three site-years is summarized in Table 9. The treatment structure was a two-way factorial (two N rates x five silicate slag rates) in a randomized complete block design with four replications. Each replication consisted of two checks (no N and Si applied), without lime and with lime at 4.5 Mg ha^−1^. The lime treatment was incorporated into the treatment structure to differentiate treatment effects from a pH effect, since slags are a potent liming agent. Two N rates of 101 and 145 kg ha^−1^ were used for this study using urea (46% N) as the N source. The 101 kg N ha^−1^ rate is the standard rate for wheat production in Louisiana, and the 145 kg N ha^−1^ rate is considered a high application rate, a rate sometimes used by farmers in the state. For each N rate, there were five silicate slag (Plant Tuff^®^, 12% Si, Dearborn, MI, USA) rates of 0, 1, 2, 4.5, and 9 Mg ha^−1^, which were equivalent to 0, 120, 240, 540, and 1080 kg Si ha^−1^, respectively. Phosphorus and potassium (K) fertilizer were applied to fields when necessary, according to the test results and recommendations of the LSU AgCenter Soil Testing and Plant Analysis Laboratory, to ensure neither nutrient was limiting in the soil. Silicate slag is the source of Si used for this study, which is a byproduct from steel industries containing 12% Si. Other known components of the slag material used in this study include Ca (23%), Mg (7%), and S (0.5%), among others. Both slag and lime treatments were broadcast applied by hand to individual plots in November of each year. Treatments were then incorporated into the soil to a depth of about 7.5 cm. Winter wheat variety Terral 8525 was drill seeded at the rate of 101 kg ha^−1^ for NERS in 2013 and 2014, and 113 kg ha^−1^ for BH in 2014 within, a week of slag and lime applications. The two N treatments were applied around Feekes growth stage (GS) 4 [59]. Urea (46%N) was broadcast applied by hand to the corresponding plot assignment. Recommended weed management practices from the LSU AgCenter were followed.

Soil samples were taken at harvest, oven-dried (Despatch LBB series; model number LBB2-18-1) at 55 °C for about 4–5 days, and then ground using a Humboldt soil grinder, and passed through a 2 mm sieve for later analysis. Extractable Si was determined by extracting soil samples with 0.5 M acetic acid (10 mL extractant and 1 g soil shaken for 1 h), followed by a modified molybdenum blue colorimetry (MBC) procedure, as outlined by Korndorfer et al. (2001) [22]. Soil inorganic N was determined by KCl extraction, followed by spectrophotometric measurement using an automated flow injection system (Lachat QuickChem 8500 series 2), similar to the method described by Keeney and Nelson (1982) [60]. Soil pH was also determined in a 1:1 ratio of soil:deionized (DI) water suspension using a pH meter. A wide range of extractable nutrients in the soil were determined by Mehlich-3 Procedure [61]. Two (2) grams of soil was extracted with 20 mL of Mehlich-3 solution (dilute acid–fluoride–EDTA solution, pH 2.5), and the extract was then analyzed using inductively coupled plasma (ICP)–optical emission spectroscopy (OEM), for several essential nutrients as well as some heavy metals (Spectro Ciros CCD ICP analyzer, SPECTRO Analytical Instruments, Kleve, Germany).

All plots were harvested using plot combine harvesters, and grain subsamples taken simultaneously were measured for moisture content, test weight, and total weight. Grain moisture content was adjusted to 12%, and yield was calculated in kg per ha^−1^.

The results on the analysis of variance (ANOVA) using PROC MIXED in SAS 9.3 on the effect of site-year, N, and Si, showed significant effect of site-year and the three-way interaction (site-year × N × Si) for most of the measured plant variables (not shown). Thus, the ANOVA was performed for each site-year to determine the effects of N, Si, and N × Si interactions on all measured parameters (SAS, 2012). The fixed effects were N, Si, and their interaction, while replication was assigned as a random effect. Treatments 1 (control) and 2 (check lime) were deleted, and the program was run as a complete factorial, in order to determine significant differences in Si treatments at each N rate. Trend analysis between grain yield and silica slag rates for each N rate of each site-year was conducted. For any significant effect at level <0.10, mean separation using LSD followed with identify treatment differences. Means of all measured parameters for all N × Si combinations were reported and compared, to identify the combination(s) which attained the optimal agronomic responses.

## 5. Conclusions

Nitrogen more consistently impacted wheat grain yield in comparison to silicate slag application across site-years. While increasing numerical trends of grain yield were observed for all Si treatment levels when compared to control treatments, significant increases were observed for 2 and 9 Mg ha^−1^ silicate slag rates for BH in 2014 only. NERS in 2013 initial soil Si levels were higher than both sites in 2014, and higher soil Si levels were recorded for soil samples collected at harvest. Nitrogen and Si both had effects on the availability of certain essential nutrients and heavy metals. The applications of silicate slag increased the 0.5 M acetic acid extractable Si and soil pH. Silicate slag applications showed effects on extractable Ca, Mg, S, Zn, NO_3_^−^, and Si in all three site-years. Silicate slag is a byproduct and contains some of the elements that were analyzed in the soil, and may partially explain some of the increases in availability observed. Although the other nutrients present in the slag were not found limiting in the soil, it is possible that the increased availability of other nutrients, whether direct effects from slag composition or indirect effects from interactions and pH, could have contributed to increases in grain yields. The application of silicate slag can influence the nitrification rate and NO_3_^−^ availability in soils from the N fertilizers by way of influencing soil pH. Because of the effectiveness of silicate slag at increasing soil Si and soil pH, it could prove even more beneficial if used as a lime treatment in place of traditional CaCO_3_. The inconsistencies observed in responses to Si treatments could be due to varying physicochemical properties of soils belonging to different series, generally identified as three major groups in a calibration study conducted for establishing fertilizer guidelines. More research will need to be conducted to further calibrate application rates for silicate slag for use in wheat production in Louisiana.

## Figures and Tables

**Table 1:** Analysis of variance on yield, nitrogen, and silicon uptake for straw and grain for each site-year.

Site-Year Factors	Yield		Nitrogen Uptake		Silicon Uptake		
	Straw	Grain	Straw	Grain	Straw	Grain	Total
***NERS 2013***							
N	0.962	0.040	0.311	0.104	0.336	0.171	0.126
Si	0.150	0.417	0.626	0.002	0.270	0.466	0.256
N × Si	0.087	0.603	0.841	0.071	0.382	0.754	0.389
***NERS 2014***							
N	0.223	0.002	0.158	<0.001	0.321	0.241	0.245
Si	0.540	0.783	0.303	0.355	0.198	0.127	0.370
N × Si	0.061	0.814	0.005	0.966	<0.001	0.042	0.003
***BH 2014***							
N	0.002	<0.001	0.003	<0.001	0.351	0.928	0.151
Si	0.619	0.014	0.459	0.003	0.352	0.568	0.303
N × Si	0.235	0.562	0.480	0.744	0.494	0.068	0.227

Notes: N—nitrogen, Si—silicon; NERS—Northeast Research Station in St. Joseph, Louisiana, USA; BH—Central Research Station in Ben Hur Farm near LSU Campus in Baton Rouge, Louisiana, USA.

**Table 2:** Mean and analysis of variance of wheat yield components, grain N and Si content, and % protein for site-year NERS 2013.

N kg ha^−1^	Slag Mg ha^−1^	Yield	Spike			Grain per Spike	1000-Grain g	N	Si	Protein
		kg ha^−1^	Count per m^2^	Weight g	Length cm			%		
101	0	4896	606 bc	1.29	7.24	24.2 bc	33.0	1.77	0.060	10.3
	1	5265	600 bc	1.43	7.42	26.0 bc	32.7	1.75	0.062	10.2
	2	5504	634 ab	1.43	7.64	26.3 bc	33.0	1.78	0.070	10.4
	4.5	5370	614 bc	1.40	7.35	26.2 bc	32.9	1.78	0.077	10.4
	9	5152	587 bc	1.28	7.42	25.7 bc	32.6	1.80	0.072	10.4
	*mean*	5237 A	608	1.36	7.41	25.7	32.8 A	1.77 B	0.068	10.3 B
145	0	4879	570 bc	1.35	7.44	28.7 ab	31.6	1.86	0.083	10.8
	1	4761	648 ab	1.36	7.31	24.0 c	30.2	1.86	0.062	10.9
	2	4861	648 ab	1.35	7.47	25.1 bc	30.2	1.87	0.077	10.9
	4.5	5209	530 c	1.36	7.39	32.1 a	31.1	1.85	0.068	10.8
	9	4991	704 a	1.27	7.14	24.3 c	29.8	1.95	0.048	11.4
	*mean*	4940 B	620	1.34	7.37	26.9	30.6 B	1.88 A	0.068	11.0 A
Analysis of variance										
N	*p*-value	0.040	0.817	0.439	0.616	0.267	<0.001	<0.001	0.888	<0.001
Si	*p*-value	0.419	0.326	0.097	0.282	0.061	0.567	0.451	0.490	0.485
N × Si	*p*-value	0.604	0.035	0.620	0.516	0.056	0.730	0.859	0.157	0.856

Notes: N—nitrogen; slag—silicate slag rate. Actual *p*-values are reported. Values with the same uppercase (N treatment) and lowercase (across N and Si combinations) letters are not significantly different according to least square difference LSD at 0.05 level of confidence.

**Table 3:** Mean and analysis of variance of wheat yield components, grain N and Si content, and % protein for site-year NERS 2014.

N kg ha^−1^	Slag Mg ha^−1^	Yield	Spike			Grain per Spike	1000-Grain g	N	Si	Protein
		kg ha^−1^	Count per m^2^	Weight g	Length cm			%		
101	0	6384	675 ab	1.49	6.83	26.1	35.9	1.70 cd	0.023	9.9 cd
	1	6517	590 b	1.53	7.02	28.6	36.0	1.69 cd	0.005	9.9 cd
	2	5987	701 ab	1.49	6.98	24.6	35.6	1.62 e	0..032	9.4 e
	4.5	6472	588 b	1.54	6.88	30.9	36.0	1.65 de	0.013	9.6 de
	9	6472	722 a	1.51	7.31	24.3	36.8	1.65 de	0.008	9.6 de
	*mean*	6317 B	655	1.51	7.01	26.9	36.1	1.66 B	0.016	9.7 B
145	0	6931	685 ab	1.62	7.34	28.6	35.8	1.76 bc	0.012	10.2 bc
	1	6790	660 ab	1.54	7.19	29.3	35.7	1.87 a	0.050	10.9 a
	2	6971	678 ab	1.50	6.92	29.5	35.2	1.75 bc	0.028	10.2 bc
	4.5	7198	757 a	1.65	7.44	27.2	35.6	1.79 b	0.023	10.4 b
	9	6868	592 b	1.48	7.06	29.7	36.1	1.75 bc	0.005	10.2 bc
	*mean*	6952 A	674	1.56	7.19	28.9	35.7	1.78 A	0.024	10.4 A
Analysis of variance										
N	*p*-value	0.002	0.556	0.366	0.134	0.179	0.124	<0.001	0.347	<0.001
Si	*p*-value	0.783	0.753	0.672	0.723	0.796	0.143	0.023	0.311	0.023
N × Si	*p*-value	0.814	0.080	0.824	0.152	0.258	0.954	0.364	0.133	0.367

Notes: N—nitrogen; slag—silicate slag rate. Actual *p*-values are reported. Values with the same uppercase (N treatment) and lowercase (across N and Si combinations) letters are not significantly different according to LSD at 0.05 level of confidence.

**Table 4:** Mean and analysis of variance of wheat yield components, grain N and Si content, and % protein for site-year BH 2014.

N kg ha^−1^	Slag Mg ha^−1^	Yield	Spike			Grain per Spike	1000-Grain g	N	Si	Protein
		kg ha^−1^	Count per m^2^	Weight g	Length cm			%		
101	0	5177 c	500	1.76	7.14	33.4	36.8	1.47 e	0.062	8.6 e
	1	5613 c	466	1.74	7.10	30.6	37.0	1.48 e	0.048	8.6 e
	2	5674 c	512	1.81	7.36	30.4	37.0	1.49 de	0.078	8.7 de
	4.5	5472 c	514	1.76	7.50	29.5	37.6	1.49 de	0.058	8.7 de
	9	6492 ab	458	1.83	7.48	39.8	37.7	1.61 abc	0.042	9.4 abc
	*mean*	5685 B	490 B	1.78	7.32	32.8	37.2	1.51 B	0.058	8.8 B
145	0	6519 ab	553	1.82	7.39	32.0	37.4	1.57 bc	0.048	9.2 bc
	1	6482 b	637	1.79	7.68	28.2	37.5	1.62 ab	0.052	9.5 ab
	2	6594 ab	650	1.75	7.75	29.0	37.0	1.55 cd	0.042	9.0 cd
	4.5	6824 a	512	1.83	7.50	33.4	38.2	1.58 bc	0.045	9.2 bc
	9	7112 a	571	1.75	7.50	30.5	37.4	1.68 a	0.050	9.8 a
	*mean*	6706 A	585 A	1.79	7.56	30.6	37.5	1.60 A	0.048	9.3 A
Analysis of variance										
N	*p*-value	<0.001	0.002	0.836	0.103	0.228	0.136	<0.001	0.109	<0.001
Si	*p*-value	0.014	0.557	0.996	0.736	0.252	0.104	<0.001	0.665	<0.001
N × Si	*p*-value	0.562	0.288	0.740	0.699	0.290	0.564	0.614	0.195	0.608

N—nitrogen; slag—silicate slag rate. Actual *p*-values are reported. Values with the same uppercase (N treatment) and lowercase (across N and Si combinations) letters are not significantly different according to LSD at 0.05 level of confidence.

**Table 5:** Mean and analysis of variance on soil pH and nutrient content of soil samples collected at harvest, NERS 2013.

N kg ha^−1^	Slag Mg ha^−1^	Soil	Nutrient Content, mg kg^−1^								
		pH	Si	NH_4_	NO_3_	P	Ca	Mg	S	Fe	Zn
0	0	5.6	68	9.9	2.6	38	2002	494	8.4	376	2.8
0	0 + lime	6.4	87	9.9	3.6	34	2359	515	8.6	316	2.9
101	0	5.7	62	9.8	2.9	31	1968	492	7.5	336	2.7
	1	5.7	60	9.6	3.5	33	2079	516	8.3	354	2.7
	2	6.0	83	9.8	3.3	31	2124	516	8.3	334	3.0
	4.5	6.3	118	10.2	4.8	35	2472	568	10.0	331	3.4
	9	6.5	138	10.5	4.9	37	2447	552	11.8	345	3.2
145	0	5.4	58	9.7	3.3	35	1987	479	8.4	389	2.9
	1	5.6	64	10.2	4.3	39	2070	494	9.2	375	3.0
	2	6.0	78	9.0	4.1	35	2242	542	9.0	351	3.2
	4.5	6.2	118	10.0	4.7	38	2343	537	9.4	350	3.1
	9	7	144	10.5	7.6	36	2645	592	11.2	315	3.1
Analysis of Variance											
N	*p*-value	0.519	0.966	0.276	0.505	0.0765	0.545	0.979	0.612	0.094	0.109
Si	*p*-value	<0.001	<0.001	0.488	<0.001	0.433	<0.001	<0.001	0.001	0.095	<0.001
N × Si	*p*-value	0.142	0.978	0.984	0.620	0.755	0.0561	0.111	0.699	0.108	0.851

**Table 6:** Mean and analysis of variance on soil pH and nutrient content of soil samples collected at harvest, NERS 2014.

N kg ha^−1^	Slag Mg ha^−1^	Soil	Nutrient Content, mg kg^−1^					
		pH	Si	NH_4_	NO_3_	Ca	Mg	Zn
0	0	5.1	36	15.3	1.3	2422	603	3.4
0	0 + lime	5.7	62	14.2	2.2	3107	668	3.9
101	0	4.9	39	15.5	2.8	2616	630	3.6
	1	5.0	51	14.2	2.0	2493	614	3.6
	2	5.2	60	14.7	3.0	2774	661	3.8
	4.5	5.1	58	15.0	3.7	2854	666	4.0
	9	5.4	93	15.2	3.9	3132	710	4.6
145	0	4.8	40	14.3	2.1	2511	609	3.3
	1	4.9	49	15.4	3.6	2576	632	3.4
	2	4.9	52	15.7	3.6	2675	636	3.6
	4.5	5.0	60	16.8	4.7	2751	651	4.0
	9	5.5	82	14.7	3.8	2988	688	4.1
Analysis of Variance								
N Effect	*p*-value	0.504	0.431	0.201	0.928	0.433	0.467	0.120
Si Effect	*p*-value	0.010	<0.001	0.030	0.919	0.009	0.055	0.001
N × Si Effect	*p*-value	0.766	0.806	0.348	0.929	0.944	0.934	0.779

**Table 7:** Mean and analysis of variance on soil pH and nutrient content of soil samples collected at harvest, BH 2014.

N kg ha^−1^	Slag Mg ha^−1^	Soil	Nutrient Content, mg kg^−1^								
		pH	Si	NH_4_	NO_3_	Ca	Mg	S	Cu	Mn	Zn
0	0	5.6	17	8.1	1.0	1713	306	7.5	1.8	54	1.7
0	0 + lime	6.1	35	6.4	1.8	2043	333	7.2	1.9	57	1.4
101	0	5.6	29	6.5	0.6	1743	306	5.1	1.9	64	1.4
	1	5.9	27	6.4	1.0	1856	322	6.0	1.9	63	1.5
	2	5.9	30	6.5	1.0	1864	328	5.7	1.8	65	1.5
	4.5	6.2	38	6.9	1.1	1980	348	7.5	1.9	68	1.8
	9	6.5	56	6.8	1.3	2254	383	9.1	1.9	72	2.0
145	0	5.6	30	6.7	0.8	1771	308	5.3	1.8	63	1.5
	1	5.9	27	6.7	0.9	1909	323	5.6	2.0	64	1.8
	2	5.8	30	6.6	1.2	1862	335	6.5	1.8	70	1.5
	4.5	5.9	45	6.7	1.3	2082	357	7.6	2.0	67	2.0
	9	6.5	95	7.8	1.8	2215	385	8.8	2.0	74	2.1
Analysis of Variance											
N Effect	*p*-value	0.676	0.066	0.242	0.036	0.491	0.509	0.914	0.816	0.467	0.115
Si Effect	*p*-value	0.006	<0.001	0.288	0.002	<0.001	<0.001	<0.001	0.054	0.009	<0.001
N × Si Effect	*p*-value	0.947	0.082	0.684	0.439	0.846	0.991	0.889	0.900	0.772	0.738

**Table 8:** Record of trial establishment and field activities.

Location	Year ^†^	Site-Year	Date			
			Si Application	Planting	N Application	Harvest
St. Joseph	2012	NERS 2013	9-November-2012	9-November-2012	1-February-2013	7-June-2013
St. Joseph	2013	NERS 2014	14-November-2013	16-November-2013	21-February-2014	6-June-2014
Ben Hur	2013	BH 2014	8-November-2013	11-November-2013	22-February-2014	22-May-2014

^†^ Year that the trial was established.

**Table 9:** Chemical properties and textural class of initial soil samples collected from all the sites.

Site-Year		Extractable Nutrients ^†^, mg kg^−1^							Total ^§^, %		OM ^¶^, %	Textural Class	
	Si ^‡^	P	K	Ca	Mg	S	Cu	Zn	N	C			pH
NERS 2013	62	138	401	2136	604	133	3.2	2.5	0.14	1.2	2.3	silt loam	5.66
NERS 2014	49	40	309	2212	625	20	3.3	1.9	0.16	1.3	2.1	silt loam	5.27
BH 2014	48	35	146	1833	481	17	1.8	1.0	0.14	0.9	1.5	silt loam	6.13

^†^ Extractable nutrients based on Mehlich-3 extraction procedure and ICP. ^‡^ Silicon determined by 0.5 M acetic acid extraction procedure and molybdenum blue colorimetry. ^§^ C and N content determined by dry combustion. ^¶^ Organic matter based on Walkley and Black method.
